# What Aspect of Dependence Does the Fagerström Test for Nicotine Dependence Measure?

**DOI:** 10.1155/2013/906276

**Published:** 2012-11-22

**Authors:** Joseph R. DiFranza, Robert J. Wellman, Judith A. Savageau, Ariel Beccia, W. W. Sanouri A. Ursprung, Robert McMillen

**Affiliations:** ^1^Department of Family Medicine and Community Health, University of Massachusetts Medical School, 55 Lake Avenue North, Worcester, MA 01655, USA; ^2^Department of Psychology and Social Science Research Center, Mississippi State University, Starkville, MS 39762, USA

## Abstract

Although the Fagerström Test for Nicotine Dependence (FTND) and the Heaviness of Smoking Index (HSI) are widely used, there is a uncertainty regarding what is measured by these scales. We examined associations between these instruments and items assessing different aspects of dependence. Adult current smokers (*n* = 422, mean age 33.3 years, 61.9% female) completed a web-based survey comprised of items related to demographics and smoking behavior plus (1) the FTND and HSI; (2) the Autonomy over Tobacco Scale (AUTOS) with subscales measuring Withdrawal, Psychological Dependence, and Cue-Induced Cravings; (3) 6 questions tapping smokers' wanting, craving, or needing experiences in response to withdrawal and the latency to each experience during abstinence; (4) 3 items concerning how smokers prepare to cope with periods of abstinence. In regression analyses the Withdrawal subscale of the AUTOS was the strongest predictor of FTND and HSI scores, followed by taking precautions not to run out of cigarettes or smoking extra to prepare for abstinence. The FTND and its six items, including the HSI, consistently showed the strongest correlations with withdrawal, suggesting that the behaviors described by the items of the FTND are primarily indicative of a difficulty maintaining abstinence because of withdrawal symptoms.

## 1. Introduction

Tools for the assessment of nicotine dependence are important for clinical research. The most widely used measure is the Fagerström Test for Nicotine Dependence (FTND, Table 1) [1]. Despite its widespread use over two decades, the literature reflects uncertainty regarding what aspects of nicotine dependence are tapped by the FTND [2]. Fagerström recently proposed changing the name to the Fagerström Test for Cigarette Dependence, to reflect both the instrument's concentration on cigarette smoking and general understanding that tobacco dependence is driven by factors in addition to nicotine [3].

The FTND correlates poorly with a diagnosis of nicotine dependence based on the International Classification of Diseases-10 (*r* = .32), the Diagnostic and Statistical Manual-III (*r* = .24), and the DSM-IV (*r* = .32) [4]. It correlates well with CO and cotinine levels, but these are not themselves specific measures of addiction [5–7]. The FTND correlates with various withdrawal symptoms [5, 7], strength of urges to smoke [8], and self-rated addiction [4, 9, 10]. Moolchan et al. suggested that the FTND taps mainly into nicotine withdrawal [9].

There has been a recent interest in the Heaviness of Smoking Index (HSI), which is made up of just items 1 and 4 from the FTND [11]. In some studies the HSI has been shown to predict smoking cessation [12, 13]. Our literature search failed to identify any study comparing the FTND or HSI to an array of indicators to identify the specific aspects of dependence to which they most closely relate.

Our purpose was to administer the FTND with a battery of other measures to obtain insight into what aspects of tobacco addiction it taps. We hypothesize that the behaviors described by the items in the FTND are primarily indicative of difficulty maintaining abstinence because of withdrawal symptoms.

## 2. Method

### 2.1. Participant Characteristics

The survey was completed by 422 current smokers. Their mean (M) age was 33.3 years (standard deviation (SD) = 13.7); 61.9% were female, and they were overwhelmingly white-non-Hispanic (86.8%); 4.3% were Hispanic, 3.6% black, 2.4% Asian, 0.5% Native American or Pacific Islander, and 2.4% were of mixed race. Their lifetime duration of smoking ranged from <1 month to 64 years, and their current frequency of smoking ranged from an average of less than once per month to daily smoking (42.8% were nondaily smokers), 11.6% smoked within 5 minutes of awakening, 25.4% within 6–30 minutes, 14.0% within 31–60 minutes, and 49.1% waited more than 60 minutes to smoke their first cigarette. Their average cigarette consumption ranged from <1 to 50 per day (*M* = 8.25, SD = 7.76); 39.9% smoked 1–5 cigarettes/day, 27.7% smoked 6–10 cigarettes/day, and 32.4% smoked >10 cigarettes/day.

### 2.2. Sampling Procedures

Participants were recruited to complete a web-based survey via postings on the website of a major health care organization in central Massachusetts, postings on Craigslist (http://boston.craigslist.org/), and a single email invitation to the students, faculty, and staff of a central Massachusetts university and students of six universities in Mississippi that participate in a research network of the Social Science Research Center at Mississippi State University. The study was approved by the Institutional Review Boards of the University of Massachusetts Medical School, Fitchburg State University, and Mississippi State University.

The announcements asked potential participants if they had smoked at least one cigarette in the preceding month and if they wished to participate in “a research study about the experiences people have when they smoke.” They were provided with a link that directed them to the consent page of the survey. Upon reading a description of the survey, respondents attested that they were at least 18 years of age, currently smoked cigarettes, and agreed to participate. The survey could be completed in less than 10 minutes (see [14] for details).

### 2.3. Survey Design and Administration

In addition to the FTND, the survey contained items related to (1) demographics (age and sex); (2) smoking behavior, including information about current and lifetime cigarette consumption, lifetime duration of smoking and the duration of the longest period of complete abstinence; (3) the Autonomy over Tobacco Scale (AUTOS) [15].

The AUTOS is a 12-item measure of the degree to which smokers have lost autonomy over their use of tobacco (Table 2). We chose the AUTOS because it has three subscales which provide independent assessments of the degree to which Withdrawal, Psychological Dependence on cigarettes, and experiences with Cue-Induced Cravings to smoke contribute to a smoker's dependence on nicotine. It has excellent internal (*α* =  .91–.97) and retest reliability (*r* = .95) [15–17].

As Moolchan and others (2002) have suggested that the FTND taps primarily into withdrawal, we included several additional items to assess the desire to smoke that is triggered by withdrawal [9, 18, 19]. For this purpose we used standardized items shown to be valid and reliable indicators [20–22]. Prior research has established operational definitions for the terms “wanting”, “craving”, and “needing” within the context of nicotine withdrawal [20, 23]. The presence of “wanting” was defined as the endorsement of the following item “If I go too long without smoking the first thing I will notice is a mild desire to smoke that I can ignore.” “Craving” was defined as the endorsement of the item “If I go too long without smoking, the desire for a cigarette becomes so strong that it is hard to ignore and it interrupts my thinking,” and “needing” was defined as endorsement of the item “If I go too long without smoking I just can't function right, and I know I will have to smoke just to feel normal again.” The duration of abstinence that precedes the onset of these withdrawal symptoms is termed the latency. Each item assessing wanting, craving, and needing was followed by a question assessing the latency; that is, how long the smoker could go without smoking before experiencing the symptom. These measures have been validated [16, 18, 19, 24]. Wanting, craving, and needing always develop in that order as dependence progresses and appear in that order during abstinence [20, 25]. For each individual, the latency to wanting is always shorter than the latency to craving, but the population mean for the latency to wanting may be longer because latencies shorten over time and novice smokers may not contribute data for the calculation of the mean latency to craving because they have not experienced this symptom [22].

As some FTND items assess how the smoker handles periods of abstinence (after an overnight abstinence, or when smoking is forbidden), we thought it would be useful to include items assessing how the smoker prepares to cope with abstinence: (1) “I am careful not to run out. I make sure I have enough cigarettes for the next morning,” (2) “If I know I am going to be in a situation where I can't smoke, I will smoke extra to prepare myself,” and (3) “To some degree I have to plan my schedule around when I will be able to smoke.” Response options for statement 1 were *describes me not at all*, *describes me a little*, *describes me pretty well*, and *describes me very well*, while those for statements 2 and 3 were dichotomous (*yes/no*). Preliminary data analysis revealed that responses to statement 1 were highly skewed; 84% of respondents, chose the lowest two response categories. For consistency with statements 2 and 3, therefore, we scored statement 1 as *not at all* versus *at least a little*.

### 2.4. Data Analyses

The survey format required participants to complete each item (other than those assessing demographics) before continuing to the next; therefore, only completed surveys were retained for analysis. The standard scoring was used for the AUTOS, FTND, and HSI (Table 1), except that item 2 on the FTND (“Do you find it difficult to refrain from smoking in places where it is forbidden?”) was reworded as “Have you ever found it hard to keep from smoking in places where you are not supposed to?” The potential range for the FTND is 0–10, for the HSI 0–6, and for the AUTOS 0–36. Internal reliability was calculated for each measure using Cronbach's *α*.

Our objective was to determine what aspects of dependence the FTND, the HSI, and the individual items measure. We took two approaches. First, we calculated Spearman rho correlations (*r*_*s*_) between the FTND, the HSI, and the 6 individual FTND items and (1) the AUTOS subscale scores For Withdrawal, Psychological Dependence, and Cue-Induced Cravings to smoke; (2) the latencies to wanting, craving, and needing; (3) the items concerning how individuals handle abstinence situations. We assessed differences between correlations using the Meng et al. [26] extension of Fisher's *r* to *z*′ transformations for correlated correlations. 

Second, we performed separate stepwise linear regression analyses using the FTND score and the HSI score as continuous outcome variables. Independent variables included age, gender, the three subscale scores from the AUTOS measuring Withdrawal, Psychological Dependence, and Cue-Induced Cravings to smoke, the three items assessing how the smoker prepares to cope with abstinence, the percent of time that cigarettes were smoked out of need, and the duration of smoking in years. A large proportion of our sample was comprised of nondaily smokers, whose scores on the FTND and HSI are likely to be far lower than those of daily smokers. Thus, we ran separate regression analyses for nondaily and daily smokers, and we ran separate models with and without forcing age and gender into the model. SPSS/PASW V17.0 was used for the data analyses. A *P* value of  .05 was used as a test of significance.

## 3. Results

### 3.1. Measures

Scores on the measures were FTND (*M* = 2.5, range = 0–9, SD = 2.5), HSI (*M* = 1.3, range = 0–6, SD = 1.5), and AUTOS (*M* = 14.0, range = 0–36, SD = 9.5). Daily smokers were far more likely than nondaily smokers to endorse the three items regarding preparation for abstinence. Whereas 52.7% of daily smokers reported having to plan their schedules around smoking, only 11.6% of nondaily smokers did so (*z* = 8.16, *P* < .001). Similarly, 74.7% of daily smokers, compared with only 8.9% of nondaily smokers, reported being careful not to run out of cigarettes (*z* = 13.39, *P* < .001). Finally, 46.3% of daily smokers, compared with only 8.8% of nondaily smokers, reported smoking extra to prepare for a situation in which they could not smoke (*z* = 8.28, *P* < .001). The mean latency to wanting was 144 hours (range = 0.5–726, SD = 239), the mean latency to craving was 48.6 hours (range = 0.1–726, SD = 120), and the mean latency to needing was 51.2 hours (range = 0.1–726, SD = 123).

Interitem correlations for the FTND ranged from 0.18 to 0.49. Cronbach's *α* = .73 for the FTND,  .57 for the HSI,  .94 for the AUTOS,  .93 for the Withdrawal subscale,  .77 for the Psychological Dependence subscale, and  .81 for the Cue-Induced Cravings subscale. The modest internal reliability of the HSI limits how strongly it can correlate with other measures.

### 3.2. Correlations

Table 3 presents correlations between the FTND and HSI and other measures. The FTND correlated moderately with the AUTOS subscales, but better with Withdrawal (*r*_*s*_ = .72) than with Psychological Dependence (*r*_*s*_ = .64, *z* = 3.79, *P* < .001) or Cue-Induced Cravings (*r*_*s*_ = .63, *z* = 4.23, *P* < .001). The HSI also correlated better with Withdrawal (*r*_*s*_ = .69) than Psychological Dependence (*r*_*s*_ = .62, *z* = 3.20, *P* = .001), or Cue-Induced Cravings (*r*_*s*_ = .60, *z* = 4.06, *P* < .001).

As expected, the correlations between the latencies and the FTND and HSI were negative (i.e., higher FTND/HSI scores were associated with shorter latencies). The strongest correlation was with the latency to wanting. With respect to the correlations between the instruments and the items assessing preparation for a period of abstinence, the strongest was with saving cigarettes for the next morning (FTND: *r*_*s*_ = .70; HSI: *r*_*s*_ = .68).

Item 1 (first morning cigarette) correlated most strongly with the AUTOS Withdrawal subscale (*r*_*s*_ = .66) and “I am careful not to run out. I make sure I have enough cigarettes for the next morning” (*r*_*s*_ = .67). Item 2 (hard to refrain) correlated most strongly with the Withdrawal subscale. Item 3 (which cigarette would you hate to give up) showed relatively low correlations with all items but correlated most strongly with the latency to wanting (*r*_*s*_ = −.37). Item 4 (daily consumption) correlated most strongly with the AUTOS Withdrawal subscale (*r*_*s*_ = .49) and also correlated with the latency to wanting (*r*_*s*_ = −.43). Item 5 (smoking more in the morning) showed relatively low correlations overall but correlated most strongly with the AUTOS Withdrawal subscale (*r*_*s*_ = .36). Item 6 (smoking when ill) correlated most strongly with the AUTOS Withdrawal subscale (*r*_*s*_ = .47).

### 3.3. Linear Regressions

Table 4 presents means, standard deviations, and intercorrelations of the FTND, HSI, and predictor variables for nondaily and daily smokers.  Table 5 presents the summary results of the linear regressions. Gender did not predict either FTND or HSI scores, while age was a significant predictor of the HSI for daily smokers.

For nondaily smokers, controlling for age and gender, FTND scores were predicted by the Withdrawal subscale, “I am careful not to run out,” and “I smoke extra to prepare myself.” HSI scores were predicted by the Withdrawal subscale, “I am careful not to run out,” and the duration of smoking. For daily smokers, controlling for age and gender, FTND scores were predicted by the Withdrawal subscale, “I smoke extra to prepare myself,” percentage of time smoked out of need, “I am careful not to run out,” and duration of smoking. HSI scores were predicted by age, the Withdrawal subscale, “I am careful not to run out,” and “I smoke extra to prepare myself.”

## 4. Discussion

Our purpose was to identify the aspects of nicotine dependence with which the FTND correlates best. We examined correlations between the FTND, and a range of items assessing different aspects of dependence. In accordance with our hypothesis and with the initial intention of the scale [27], the HSI, the FTND and the six individual items consistently showed the strongest correlations with nicotine withdrawal. An examination of FTND/HSI item content also supports a conclusion that the FTND and HSI are measures of the degree to which smokers experience nicotine withdrawal. The regression analyses reveal that this relationship is robust, as it holds for both nondaily and daily smokers.

When novice smokers first experience withdrawal symptoms, smoking a single cigarette relieves those symptoms and postpones their reappearance for several days [21, 22]. When this is the case, smokers might have an FTND or HSI score of zero as they smoke infrequently and have no trouble abstaining from smoking for days at a time. However, as dependence grows, the latency to the onset of withdrawal becomes shorter [22]. When the latency to wanting becomes shorter than the time spent in bed, the smoker awakens in a state of withdrawal and feels the need to smoke upon arising. Such smokers may get into the habit of saving cigarettes for the morning. FTND/HSI item 1, time to first cigarette, correlated with the latency to wanting and saving a cigarette for morning. The shorter the latency to wanting is, the sooner the smoker is likely to smoke after arising. Over a number of years, the latency to wanting can shorten from days to minutes [22]. As the latency to wanting shortens, smokers find they cannot space their cigarettes as far apart as they have been accustomed. Smokers do not have to wait until they experience withdrawal symptoms to smoke. In the current study, the number of cigarettes smoked per day (FTND item 4, HSI item 2) correlated with the latency to wanting; that is, the shorter the latency is, the more cigarettes are smoked per day. This is consistent with prior studies [18, 19, 28]. The connection between the latency to wanting and the number of cigarettes smoked per day provides a physiologic explanation for why daily consumption is an indirect measure of addiction.

When the latency to wanting shortens to the point where the length of time spent in a place where smoking is forbidden is shorter than the latency, the smoker will experience difficulty refraining from smoking (FTND item 2). This item correlated with the latency to wanting. When the latency to wanting is shorter than the time spent in bed, the first morning cigarette will be the hardest to give up (FTND item 3) because it is the one that provides relief from overnight withdrawal. Of the 19 items we compared it to, FTND item 3 correlated best with the latency to wanting. When a smoker is sick in bed (FTND item 6), nicotine withdrawal can only make them feel worse. FTND item 6 correlated best with the AUTOS Withdrawal scale score. Presumably, smokers smoke more cigarettes in the morning because they are in withdrawal after an overnight abstinence. The highest correlation for smoking more in the morning (FTND item 5) was with the AUTOS Withdrawal subscale. Although the FTND does not ask about withdrawal symptoms directly, each of the FTND items appears to tap into some aspect of the smoker's behavior that is influenced by nicotine withdrawal. The HSI correlated with all indicators in a manner very similar to the FTND, but had poorer internal reliability, as might be expected from such a short instrument.

The FTND and HSI correlated moderately with Psychological Dependence and Cue-Induced Craving. As seen in Table 4, the 3 subscales of the AUTOS correlate moderately well with each other so it is to be expected that anything that correlates well with the Withdrawal subscale will correlate to some degree with the other subscales as well. 

Study strengths include the sample size, the wide age range of the sample, the use of a nontreatment seeking community sample, the inclusion of individuals with a wide range of tobacco use experience, and the use of multiple comparators. An additional strength is the use of measures (the AUTOS and the latencies) that have been validated for adult smokers. Although correlates of FTND item 1 (time to first cigarette) have been examined [11, 29] to our knowledge, this is the first study to investigate the correlates of all of the FTND items.

Study limitations include a primarily white sample, the lack of information about the socioeconomic status of subjects, and the use of a convenience sample recruited through the internet. It should be noted that comparisons of web-based studies with more typical laboratory or field-based methods have demonstrated both a high degree of consistency in the data collected and a relative diversity in sample composition [30, 31]. Online methods of assessing smoking behavior and related constructs were recently found to be highly reliable and valid in a nationally representative sample of young adults in the USA [32]. An important limitation is that there are aspects of dependence that are assessed by instruments such as the Wisconsin Inventory of Smoking Dependence Motives and the Nicotine Dependence Syndrome Scale that were not covered by our measures [33, 34]. As we were relying on unpaid volunteers, there are limits to survey length. Our study should be replicated with the possible inclusion of additional measures.

Another important caveat is that a correlation between the FTND and another measure does not prove that they measure the same thing. Indeed, the FTND correlated significantly with every measure included in this study, as would be expected if all of the measures tap into nicotine dependence. Nevertheless, our data suggest that the FTND is an instrument that primarily taps into behaviors that reflect how smokers cope with nicotine withdrawal.

## Figures and Tables

**Table 1:** The Fagerström Test for Nicotine Dependence (FTND).

Item	Questions	Answers	Points
1∗	How soon after you wake up do you smoke your first cigarette?	Within 5 minutes	3
		6–30 minutes	2
		31–60 minutes	1
		After 60 minutes	0
2	Do you find it difficult to refrain from smoking in places where it is forbidden for example church, at the library, in the cinema, and so on?	Yes	1
		No	0
3	Which cigarette would you hate to give up?	The first one in the morning	1
		All others	0
4∗	How many cigarettes do you smoke a day?	10 or less	0
		11–20	1
		21–30	2
		31 or more	3
5	Do you smoke more during the first two hours than during the rest of the day?	Yes	1
		No	0
6	Do you smoke if you are so ill that you are in bed most of the day?	Yes	1
		No	0

∗Items 1 and 4 comprise the HSI (Heaviness of Smoking Index).

**Table 2:** The Autonomy Over Tobacco Scale.

	This statement describes me…			
Withdrawal subscale	Not at all	A little	Pretty well	Very well
When I go too long without a cigarette I get nervous or anxious	0	1	2	3
When I go too long without a cigarette I lose my temper more easily	0	1	2	3
When I go too long without a cigarette I get strong urges that are hard to get rid of	0	1	2	3
When I go too long without a cigarette I get impatient	0	1	2	3
Psychological Dependence subscale				
I would go crazy if I could not smoke	0	1	2	3
I rely on smoking to deal with stress	0	1	2	3
I rely on smoking to take my mind off being bored	0	1	2	3
I rely on smoking to focus my attention	0	1	2	3
Cue-Induced Craving subscale				
After eating I want a cigarette	0	1	2	3
When I smell cigarette smoke I want a cigarette	0	1	2	3
When I see other people smoking I want a cigarette	0	1	2	3
When I feel stressed I want a cigarette	0	1	2	3

**Table 3:** Correlations between the FTND and HSI and the AUTOS and items assessing preparation for abstinence and latencies to withdrawal experiences.

	FTND item number							
	1	2	3	4	5	6	FTND	HSI∗∗
AUTOS score	.66	.53	.30	.49	.36	.48	.72	.68
Withdrawal subscale	**.66**	**.53**	**.33**	**.49**	**.36**	**.47**	**.72**	**.69**
Psychological Dependence subscale	.58	.46	.26	.47	.34	.43	.64	.62
Cue-Induced Cravings to smoke subscale	.60	.47	.24	.41	.29	.43	.63	.60
I am careful not to run out. I make sure I have enough cigarettes for the next morning. (*n* = 421)	**.67**	**.43**	.33	**.49**	**.34**	**.45**	**.70**	**.68**
If I know I am going to be in a situation where I can not smoke, I will smoke extra to prepare. (*n* = 421)	.46	**.43**	.27	.37	.28	.43	.53	.48
I have to plan my schedule around smoking.	.46	.37	.22	.38	.31	.34	.52	.48
What percent of the time do you smoke because you need to at that moment?	.48	**.43**	.24	.33	.33	.38	.54	.49
Latency in hours to wanting (*n* = 303)	−.65	−.42	**−.37**	−.43	−.25	−.39	−.66	−.65
Latency in hours to craving (*n* = 235)	−.50	**−.43**	−.30	−.39	−.21	−.33	−.59	−.52
Latency in hours to needing (*n* = 169)	−.37	−.33	−.13∗	−.26	−.13∗	−.07∗	−.37	−.39

∗Not significant. All other correlations were significant at *P* ≤ .001. *n* = 422 unless otherwise indicated. The *n* for the latencies is smaller because they apply only to smokers who have experienced them. Bold values are the two strongest correlations for each column.

∗∗HSI: Heaviness of Smoking Index (items 1, 4).

**Table 4:** Means, standard deviations and intercorrelations for the FTND and HSI with predictor variables (age, AUTOS subscales, percentage of time smoked out of need, and duration of smoking) for nondaily and daily smokers.

Measure	1	2	3	4	5	6	7	8	*M *	SD
(1) FTND	—	0.68	0.12	0.45	0.39	0.38	0.25	0.08	0.69	1.17
(2) HSI	0.89	—	−0.04	0.47	0.36	0.38	0.23	−0.12	0.25	0.70
(3) Age	0.34	0.37	—	0.05	0.10	0.09	0.03	0.51	26.78	10.14
(4) Withdrawal	0.50	0.43	0.03	—	0.72	0.70	0.55	0.11	1.32	2.27
(5) Psychological Dependence	0.40	0.37	−0.06	0.66	—	0.77	0.59	0.25	1.87	2.17
(6) Cue-Induced Cravings	0.35	0.31	−0.16	0.66	0.58	—	0.55	0.28	3.59	2.93
(7) % time smoked out of need	0.43	0.32	0.16	0.45	0.36	0.29	—	0.14	19.02	27.38
(8) Duration of smoking	0.40	0.41	0.83	0.11	0.06	−0.05	0.16	—	6.20	8.89

*M *	3.80	2.13	38.15	6.18	5.39	7.92	43.80	18.65		
SD	2.32	1.40	14.00	3.46	2.73	2.48	27.02	13.57		

Note: intercorrelations for nondaily smokers (*n* = 181) and for daily smokers (*n* = 241) are presented above and below the diagonal, respectively. Means and standard deviations are presented in the columns for nondaily smokers and in the rows for daily smokers. Correlations are nonparametric (*r*_*s*_).

**Table 5:** Hierarchical multiple regression analyses predicting FTND and HSI from AUTOS subscales, percentage of time smoked out of need, duration of smoking, and items assessing preparation for abstinence in nondaily and daily smokers.

Predictor	FTND		Predictor	HSI	
	Δ*R* ^2^	β		ΔR^2^	β
	Nondaily smokers (*n* = 181)				

Step 1	.03		Step 1	.00	
Age		.07	Age		.11
Sex		.02	Sex		.01
Step 2	.33∗∗∗		Step 2	.18∗∗∗	
Withdrawal Subscale		.38∗∗∗	Withdrawal subscale		.31∗∗∗
Step 3	.08∗∗∗		Step 3	.03∗	
Careful not to run out		.24∗∗	Careful not to run out		.21∗∗
Step 4	.02∗		Step 4	.03∗	
Smoke extra to prepare		.18∗	Duration of smoking		−.20∗

Total *R* ^2^	.46∗∗∗		Total *R* ^2^	.24∗∗∗	

	Daily smokers (*n* = 241)				

Step 1	.12∗∗∗		Step 1	.14∗∗∗	
Age		.12	Age		.33∗∗∗
Sex		.07	Sex		.04
Step 2	.23∗∗∗		Step 2	.18∗∗∗	
Withdrawal Subscale		.25∗∗∗	Withdrawal subscale		.28∗∗∗
Step 3	.04∗∗∗		Step 3	.04∗∗∗	
Smoke extra to prepare		.18∗∗	Careful not to run out		.19∗∗
Step 4	.02∗∗		Step 4	.02∗	
% Time smoke out of need		.19∗∗∗	Smoke extra to prepare		.14∗
Step 5	.02∗∗				
Careful not to run out		.14∗			
Step 6	.01∗				
Duration of smoking		.20∗		—	
	—				.14∗

Total *R* ^2^	.45∗∗∗		Total *R* ^2^	.37∗∗∗	

∗ *P* < .05, ∗∗*P* ≤ .01, ∗∗∗*P* < .001.
